# Supplementary material for: Antiviral Activity of Ethyl Gallate Against Zika Virus: In Vitro and In Silico Studies
Source: Int J Mol Sci. 2025 Dec 15;26(24):12062. doi: 10.3390/ijms262412062 (PMC12733338; doi:10.3390/ijms262412062)
Supplement: Supplementary file 1 [file ijms-26-12062-s001.zip › ijms-3966964-supplementary.pdf]

a

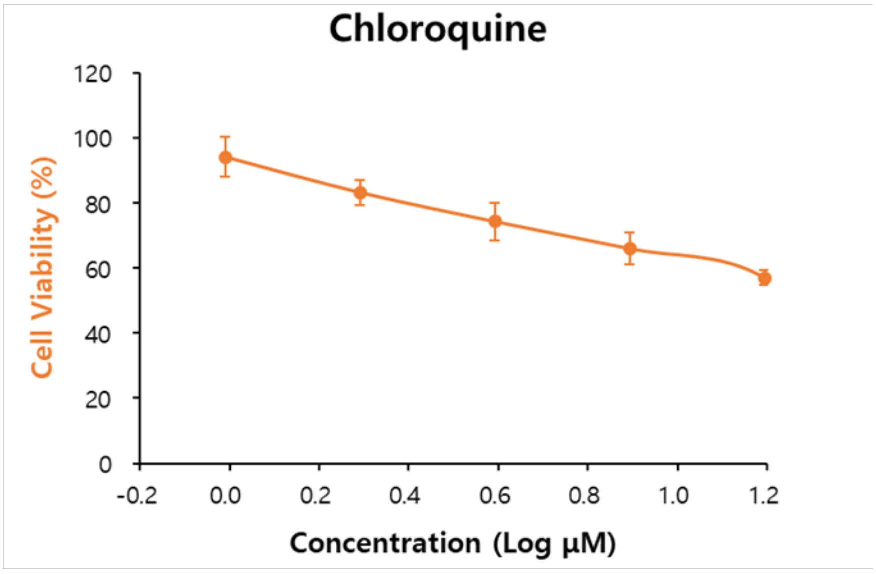

b

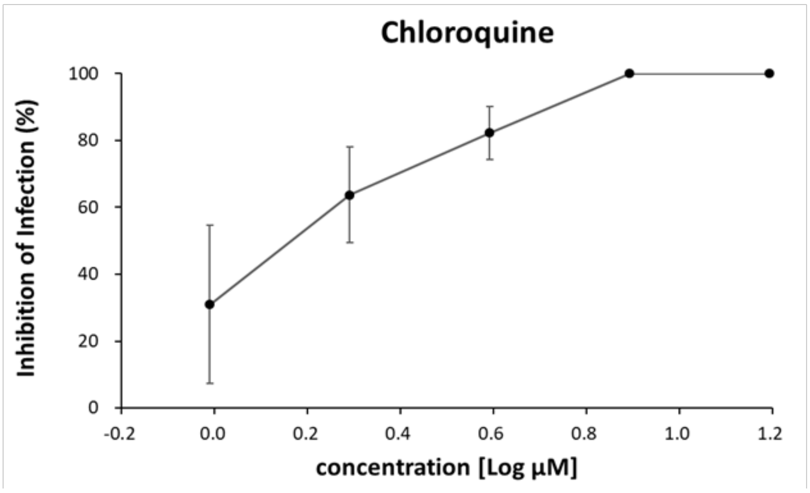

Figure S1. Cytotoxicity and antiviral activity of chloroquine in Vero E6 cells.

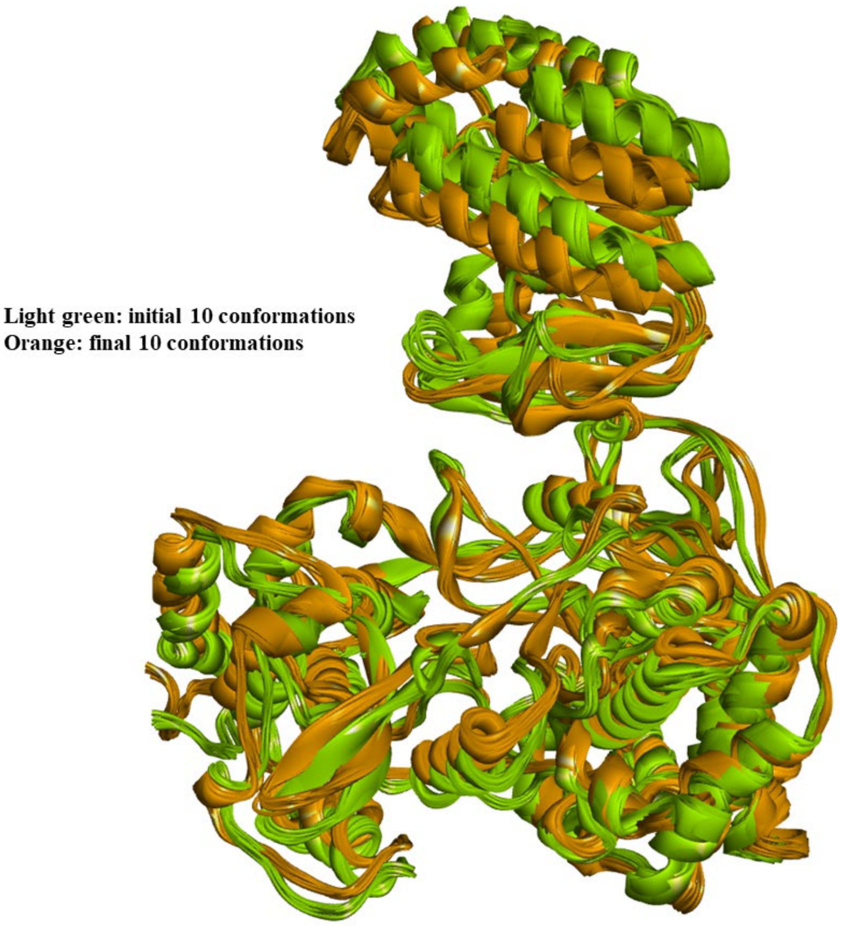

Figure S2. Conformational change of RdRp apo form during MD simulation.
